# ERα-Independent Activity of Tamoxifen-Based Transition Metal Hybrids in Triple-Negative Breast Cancer Models In Vitro and In Vivo

**DOI:** 10.3390/molecules31091376

**Published:** 2026-04-22

**Authors:** Blagoje Murganić, Tamara Krajnović, Duško Dunđerović, Aleksandr Kazimir, Nasta Tanić, Nikola Tanić, Evamarie Hey-Hawkins, Danijela Maksimović-Ivanić, Sanja Mijatović

**Affiliations:** 1 Department of Radiobiology and Molecular Genetics, Institute of Nuclear Sciences “Vinča”, National Institute of Republic of Serbia, University of Belgrade, 11351 Belgrade, Serbia; blagoje@vin.bg.ac.rs (B.M.); nastad@vin.bg.ac.rs (N.T.); 2 Department of Immunology, Institute for Biological Research “Siniša Stanković”, National Institute of the Republic of Serbia, University of Belgrade, 11108 Belgrade, Serbia; tamara.krajnovic@ibiss.bg.ac.rs (T.K.); nelamax@ibiss.bg.ac.rs (D.M.-I.); 3 Faculty of Medicine, Institute of Pathology, University of Belgrade, 11000 Belgrade, Serbia; dusko.dundjerovic@med.bg.ac.rs; 4 Institute for Drug Discovery, Faculty of Medicine, Leipzig University, 04103 Leipzig, Germany; alexandr.kazimir@uni-leipzig.de; 5 Department of Natural Sciences and Mathematics, State University of Novi Pazar, 36300 Novi Pazar, Serbia; 6 Department of Neurobiology, Institute for Biological Research “Siniša Stanković”, National Institute of the Republic of Serbia, University of Belgrade, 11108 Belgrade, Serbia; nikolata@ibiss.bg.ac.rs; 7 Institute of Inorganic Chemistry, Faculty of Chemistry and Mineralogy, Leipzig University, 04103 Leipzig, Germany; hey@uni-leipzig.de

**Keywords:** tamoxifen derivatives, tamoxifen resistance, TNBC, metallo-drugs, hybrid molecules, cancer therapy

## Abstract

Multiple studies have demonstrated that the conjugation of various metal cores to a modified tamoxifen vector amplifies its antitumor activity, rendering such engineered structures effective even in triple-negative breast cancer (TNBC), a tumor subtype traditionally considered irrelevant for endocrine therapy. With a focus on TNBC cell line, this study shows that hybrids with Pd- and Cu- in comparison to Pt-based counterparts exerted an advanced cytotoxic profile in terms of sustained cytotoxicity throughout all tested periods, well synchronized with an intensive and prolonged oxidative burst measured by 4-amino-5-methylamino-2′,7′-difluorofluorescein diacetate (DAF-FM), dihydroethidium (DHE), and dihydrorhodamine 123 (DHR-123) in the background. Translation to the orthotopic syngeneic mouse in vivo model confirmed their superiority toward Pt-based conjugates, as well as tamoxifen alone, with a more profound tumor-reducing potential of Cu-tamoxifen, which was finally restricted by its toxicity. Surprisingly, the tamoxifen vector per se, with an approx. 2-fold lower cytotoxic potential than Pt- and Cu-hybrids in vitro, showed exceptional tumor-reducing potential in vivo, profiled in the last days of the treatment period. Intensive infiltration of immune cells, preferentially lymphocytes, was observed in tumor samples from animals exposed to the tamoxifen vector, underscoring the ligand’s immune potential and again suggesting that cytotoxicity is not a measure of successful treatment.

## 1. Introduction

Breast cancer (BC) is the most common malignancy in women worldwide and is the second leading cause of cancer-related mortality globally [1,2,3]. It is a highly heterogeneous disease with distinct subtypes that differ in their genetics, clinical characteristics, and responses to therapy [1,4]. Molecular classification of breast cancer is based on the expression of three primary protein markers: estrogen receptor (ER), progesterone receptor (PR), and human epidermal growth factor receptor 2 (HER2) [1]. Luminal A (ER+/PR+/HER2− is the most frequent subtype of the approximately 50–70% of all cases of BC, characterized by slow growth, high sensitivity to hormone therapy, and the best overall prognosis [4]. Luminal B (ER+/PR+/HER2+ or −) is characterized by lower levels of hormone receptors and higher expression of proliferation markers like Ki67, resulting in a more aggressive illness than Luminal A [4]. HER2-enriched (ER−/PR−/HER2+) BC comprises 15–20% of cases. These tumors grow rapidly but are susceptible to HER2-targeted antibodies such as trastuzumab [2,4]. Finally, Triple-Negative Breast Cancer (TNBC) lacks all three receptors (ER−/PR−/HER2−) and accounts for 10–30% of human breast tumors [1,2]. TNBC is the most aggressive subtype, associated with high metastatic potential and poor short-term prognosis due to a lack of effective targeted therapies [1,2].

Tamoxifen (T) has been the drug of choice for endocrine therapy of patients with ER-positive breast cancer for more than a five decades [3,5,6]. As a selective estrogen receptor modulator (SERM), tamoxifen acts as a prodrug, undergoing hepatic metabolism by cytochrome P450 enzymes to form several active metabolites, such are 4-hydroxytamoxifen (4-OHT) and 4,4′-dihydroxytamoxifen (4,4′-diOHT) [3,7,8]. The primary target of these metabolites is estrogen receptor alpha (ERα), a ligand-dependent nuclear transcription factor [9,10]. In the presence of estradiol, activated ERα binds to specific DNA regulatory sequences, estrogen response elements (ERE), promoting the expression of genes that code for growth and proliferation factors [9]. Tamoxifen metabolites competitively inhibit this process by binding to the ligand-binding domain (LBD) of ERα, blocking the recruitment of co-activators, and thereby blocking estrogen-mediated proliferation [2,3,11]. Beyond its primary role as a modulator of the classical ERα, tamoxifen interacts with several alternative receptors and cellular pathways [12,13]. Specifically, it acts as an agonist on the ERα36 variant to promote cancer stemness, and binds to G protein-coupled estrogen receptor 1 (GPER) to mechanically reprogram the tumor microenvironment by downregulating hypoxia inducible factor 1 alpha (HIF-1A) [13,14]. Furthermore, it serves as an inverse agonist for cannabinoid receptors (CB1-R and CB2-R) and inhibits various enzymes, including protein kinase C (PKC) and matrix metalloproteinases [12,15,16]. It induces oxidative stress, and interferes with calcium homeostasis and lipid metabolism. Tamoxifen also affects multidrug resistance transporters like P-glycoprotein and can potentiate innate immunity by activating the nuclear factor erythroid 2-related factor 2n (NRF2) and Caspase-1 pathways in macrophages. These ER-independent actions have prompted the investigation of tamoxifen in ER-negative conditions, including its potential to act against breast cancer stem cells (CSCs) [12,16].

Despite the clinical success of tamoxifen, approximately 40% of patients initially responding to therapy develop acquired resistance [3], while up to 30% of ER-positive tumors display intrinsic resistance at diagnosis [17]. This phenomenon is driven by downregulation or loss of ERα, activation of compensatory signaling pathways such as PI3K/AKT/mTOR and MAPK/ERK, and expression of the ERα36 isoform, which promotes estrogen-independent growth. Consequently, tamoxifen resistance represents one of the major unresolved challenges in endocrine therapy [9,18]. These resistance mechanisms not only limit the long-term efficacy of hormone-based treatments but also emphasize the need for therapeutics that act through non-canonical, receptor-independent mechanisms—an area in which metal-based anticancer agents offer distinct and largely unexplored advantages [1,5,9,18,19,20,21]. Metal complexes are still a cornerstone of modern cancer therapy owing that to their unique chemical features—including redox activity, diverse coordination geometries, and tunable ligand-exchange kinetics—that are rarely achievable with conventional organic drugs. Although metal-based agents are employed across a broad spectrum of solid tumors, their importance is particularly pronounced in the treatment of aggressive malignancies such as TNBC, which lacks actionable molecular targets [1,5,22,23].

Platinum-based complexes, principally cisplatin, carboplatin, and oxaliplatin, remain foundational cytotoxic agents in contemporary treatment protocols for solid malignancies. These Pt(II) coordination complexes are among the most extensively used chemotherapeutic drugs worldwide and are incorporated into guideline-driven regimens for a range of solid tumors, including ovarian, testicular, lung, head and neck cancers, and others, often as backbone components of combination therapy with radiotherapy, targeted agents, or immune checkpoint inhibitors [11,20]. Rather than serving as universal monotherapies in all settings, current practice emphasizes their use within multimodal strategies designed to enhance efficacy while mitigating intrinsic and acquired resistance and dose-limiting toxicities. Namely, their potent anticancer activity arises primarily, though not exclusively, from their ability to enter the cell nucleus and form intra- and interstrand cross-links with genomic DNA, thereby disrupting DNA replication and transcription and ultimately triggering programmed cell death [24]. However, this mechanism of action, and lack of selectivity toward malignant phenotype, is tightly associated with severe systemic toxicities, most notably nephrotoxicity, as well as with the rapid emergence of acquired drug resistance [1,11,20,21,25]. Consequently, one of the major contemporary strategies in anticancer drug design focuses on improving the pharmacological profile of platinum-based agents through replacement or modification of ligand and/or the metal center within the active core. Although replacement of the platinum center represents an important tactic for improving the pharmacological profile of metal-based anticancer agents, platinum itself remains a relevant reference scaffold in the design of novel coordination compounds. Palladium (Pd) is often considered an alternative to platinum, sharing similar coordination chemistry but possessing significantly faster ligand exchange rates, which often results in decreased potency compared to cisplatin counterparts. [26,27]. However, appropriate design of the ligands for Pd complexes can enhance the activity against chemotherapy-resistant tumors and lower off-target toxicity [1,20]. Copper (Cu), an endogenous essential trace metal, is integrated into multiple physiological processes, including redox regulation, mitochondrial function, and enzymatic antioxidant defense. In contrast to non-essential heavy metals such as platinum, copper homeostasis is tightly controlled by dedicated transporters and chaperones, leading to the hypothesis that copper-based anticancer complexes may display reduced toxicity toward normal cells while retaining antitumor efficacy [20,21,28]. In addition, malignant cells often exhibit copper addiction, characterized by increased uptake and utilization of copper to support proliferation, angiogenesis, and oxidative stress adaptation, similar to the well-established iron dependence of cancer cells [29]. This supports the rationale for exploiting copper homeostasis and copper-based complexes as selective therapeutic targets in cancer. Importantly, copper complexes are particularly effective at exploiting the hypoxic environment of tumors. In these conditions, Cu(II) is reduced to Cu(I), which catalyzes the production of reactive oxygen species (ROS) to induce lethal oxidative stress and DNA damage [20,21,28].

**Scheme 1:**
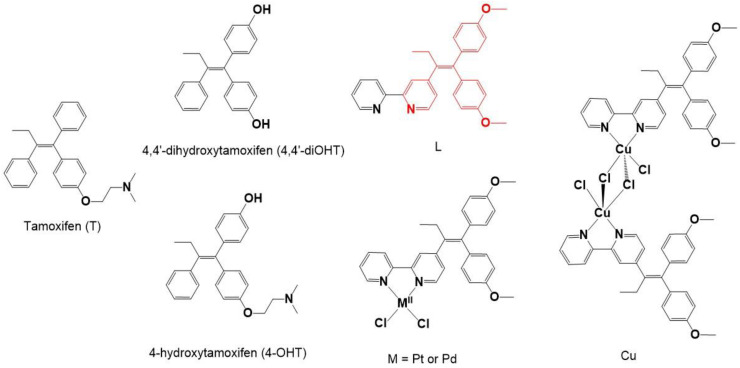
Chemical structures of tamoxifen, tamoxifen metabolites and tamoxifen-based derivatives. The tamoxifen-derived core motif is highlighted in red in ligand L.

Following the concept of a combination therapy with the aim to surmount the limitations of the above-mentioned conventional treatment and improving therapeutic efficacy, hybrid metallodrugs, which combine the targeting vector of a known ERα inhibitor–tamoxifen, with the potent cytotoxic properties of bioactive transition metals, were developed [5,6,30,31]. Compared to the classical combination therapy, where two or more agents (therapies) are administrated separately, hybrid molecules offer the advantage of simultaneous and stoichiometrically define delivery of two pharmacophores releasing dual (multi-) therapeutic activity. This structural combination enables a coordinated and potentially synergistic biological activity of the therapeutics at the target site. Consequently, hybrid metallodrugs are expected to enhance therapeutic efficacy in breast cancer treatment through the complementary action of the ER inhibitor and the transition metal component, while potentially delaying resistance development. Previously, inspired by 4-OHT and 4,4′-diOHT structural motifs we have combined tamoxifen vector with 2,2′-bypiridine resulting in 4-[1,1-bis(4-methoxyphenyl)but-1-en-2-yl]-2,2-bipyridine (L) [5,6,32]. This combination enabled incorporation of platinum-[PtCl_2_(L-κ^2^*N*,*N*′)] (PtL), palladium-[PdCl_2_(L-κ^2^*N*,*N*′)] (PdL), or copper dichloride-[CuCl(μ-Cl)(L-κ^2^*N*,*N*′)]_2_ (CuL) in L structure (Scheme 1) [5,6].

These tamoxifen-based metallodrugs integrate two pharmacologically active domains within a single chemical entity: (i) the tamoxifen scaffold, responsible for selective estrogen receptor modulation, and (ii) the metal center, which contributes additional biological activity through distinct mechanisms such as redox modulation or coordination-driven interactions. This dual-functional design is consistent with the definition of hybrid molecules as compounds combining two pharmacophores with complementary or synergistic biological functions. According to their chemical features and cellular physiology, created compounds inside of the cells probably undergo stepwise ligand exchange, redox transformations, and interactions with biological nucleophiles such as glutathione, proteins, and other donor molecules, generating a range of transient and potentially bioactive species. Therefore, their effects probably arise not only from the dual biological activity of hybrid structure, but also from its intracellularly transformed intermediates and, possibly, from partial ligand release over time. The activity of designed drugs was tested on various human cancer cell lines expressing ERα [5,6]. Building upon the initially perceived unexpectedly high sensitivity of ERα MDA-MB-231 to tested drugs, it was intriguing to examine how universal this phenomenon is for both, TNBC and cell lines derived from tumor types that are not inherently associated with hormonal activity. This approach is in line with drug repurposing concept as a highly valuable in the development of new therapeutic strategies. Concordantly, even that tamoxifen is not a drug of choice for TNBC, it exhibits properties that point to activities not directly related to ERα inhibition. 

Given the discrepancy between in vitro cultures and the complexity of living systems, for the first time, the effectiveness of such hybrid molecules has been tested in an in vivo environment using a BALB/c syngeneic orthotopic mouse breast cancer model. The obtained data highlight the potential superiority of subtle chemical interventions over cytotoxic drug-designed paradigms, emphasizing the importance of targeting the tumor microenvironmental network as an integrated system and demonstrating the therapeutic advantage of modulatory, rather than purely cytotoxic activity.

## 2. Results

### 2.1. Metal-Tamoxifen Hybrids Cytotoxicity Against Hormone-Independent Cell Lines

To investigate the cytostatic and cytotoxic properties of a tamoxifen vector conjugated with platinum, palladium or copper complexes, we selected tumor cell lines that are not suitable candidates for tamoxifen-based therapy, either due to the absence of ERα or because they do not belong to hormone-dependent neoplasms [33]. For this purpose, we employed the TNBC 4T1 cell line and two melanoma cell lines B16 and B16F10, representing different levels of low and highly invasive clones. Cells were treated with the experimental therapeutics for 72 h, and viability was assessed using two complementary assays: 3-(4,5-dimethylthiazol-2-yl)-2,5-diphenyltetrazolium bromide (MTT), which measures mitochondrial respiration, and crystal violet (CV), which quantifies total cellular DNA/RNA/protein content. As shown in Table 1 and Figure S1, all treatments produced a clear dose-dependent decrease of cell viability. 

IC_50_ values obtained by both assays revealed a pronounced sensitivity across all tested cell lines, with potencies exceeding those of the active tamoxifen metabolite used as a reference compound—often by several folds. Notably, in contrast to the reference drug, the newly designed agents displayed slightly but non-negligibly higher activity against cell lines representing more advanced tumor phenotypes. This suggests that the introduced chemical modifications may have resulted in altered mechanisms of action, potentially improving the targeting of stem-like tumor subpopulations. Among the metal-based constructs, the palladium complex exhibited slightly lower potency compared to its platinum counterparts according to the IC_50_ criterion, whereas the apparent superiority of the Cu–tamoxifen hybrid should be interpreted in light of its dimeric structure, which contains doubled tamoxifen and copper content. Namely, although CuL adopts a dimeric structure in the solid state, it dissociates in solution under experimental conditions to yield monomeric species with a 1:1 metal-to-ligand ratio [6]. Importantly, the tamoxifen ligand used to generate the metal–tamoxifen hybrids demonstrated considerable intrinsic cytotoxicity, indicating that the modified tamoxifen scaffold itself retains significant off-target-based anticancer potential (Table 1, Figure S1).

### 2.2. Basic Mechanism of Metal-Tamoxifen Hybrids Off-Target Cytotoxic Action

To elucidate the mechanism underlying the viability reduction induced by tamoxifen-metal complex hybrids—cell proliferation, presence of apoptotic/necrotic/autophagic cell death, total caspase activity, as well as production of reactive oxygen and nitrogen species (ROS/RNS) were evaluated using flow cytometry.

**Figure 1:**
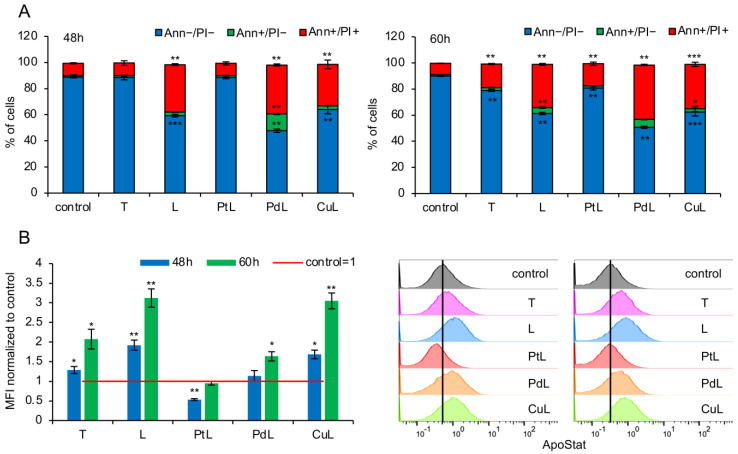
L, PtL, PdL, and CuL induced caspase-dependent apoptosis of 4T1 cells. Cells were exposed to an IC_50_ concentration of experimental compounds for 48 h and 60 h, and analyzed by flow cytometry: (**A**) apoptosis detection (Annexin V-FITC/PI staining); (**B**) caspase activity (ApoStat staining). * *p* > 0.05, ** *p* > 0.01, *** *p* > 0.001, compared to control.

Assessment of the proliferation rate within 72 h using the CFSE staining protocol revealed a moderate delay in cell division only in cultures treated with IC_50_ concentrations of the L and the Pd–tamoxifen complex. In contrast, the remaining compounds did not significantly impair proliferation during the same time period. These findings indicate that impaired cell division is not the principal cause of reduced viability in cultures exposed to the experimental therapeutics, suggesting instead that cell death induction is the dominant mode of action of these newly designed compounds (Figure S2). Annexin V-FITC/PI dual staining, one of the most subtle methods for detecting early and late apoptosis, demonstrated that 4T1 cells treated with IC_50_ concentrations of all hybrids for 48 h accumulated substantial levels of apoptotic cells (Figure 1A). The strongest effects were observed in cultures exposed to L, Cu-, and Pd-based complexes. Prolonged time of incubation for an additional 12 h resulted in a modest increase of apoptosis detected in cells treated with the active tamoxifen metabolite and its hybrid Pt analogue. However, these levels still remained markedly lower than those induced by L, Pd-, and Cu-containing conjugates. Extended incubation confirmed a persistently high apoptotic response in the latter group (Figure 1A). These distinct temporal patterns indicate that intracellular accumulation, activity, and molecular target engagement of the compounds depend strongly on the incorporated metal. This conclusion is supported by nuclear morphology mapping of fixed 4T1 cells using propidium iodide staining (PI), which visualized characteristic apoptotic alterations in chromatin structure (Figure S3). Together with apoptotic related changes in nuclear morphology, only in cultures exposed to tamoxifen vector without metal subunit, significantly engorged senescent like nuclei were observed, indicating well synchronised cytostatic and cytotoxic activities (Figure S3). In this light, the approximately two-fold higher IC_50_ values determined by the CV assay of tamoxifen ligand relative to the metal-based hybrids, may partially reflect the elevated nucleic acid and/or protein content of enlarged 4T1 cells, which can further lead to an overestimation of viable cell mass.

The total caspase activity, measured using the ApoStat assay, generally correlated with the apoptotic levels across all treatments (Figure 1B). An exception was observed with the Pt–tamoxifen complex, which showed a pronounced but transient reduction in caspase within the examined timeframe (Figure 1B).

**Figure 2:**
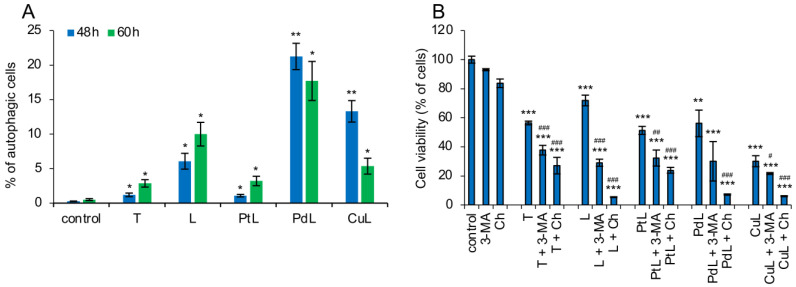
Treatment with all compounds led to the induction of autophagy. (**A**) 4T1 cells were treated with IC_50_ concentrations of experimental agents for 48 h and 60 h, stained with acridine orange dye and subsequently analyzed by flow cytometry; (**B**) The viability of 4T1 cells treated with IC_50_ concentrations of the substances for 72 h, in the presence or absence of autophagy inhibitors, 3-methyladenine (3-MA) and chloroquine (Ch), measured by crystal violet assay.* or # *p* > 0.05, ** or ## *p* > 0.01, *** or ### *p* > 0.001 (* compared to control, # compared to treatment).

In parallel, supravital acridine orange staining revealed robust autophagosome formation in cells treated with L, Pd-, and Cu-based hybrids, following the trend Pd > Cu > L. From 48 to 60 h, autophagic activity increased dramatically, reaching up to a 20-fold elevation relative to control cultures (Figure 2A). This strong autophagic response was closely associated with the extensive apoptosis triggered by the treatment. A similar but significantly weaker correlation between autophagy and apoptosis was observed in cultures treated with the active tamoxifen metabolite T and the Pt–tamoxifen hybrid, consistent with their lower apoptotic rate and slower accumulation of apoptotic cells at 48 and 60 h (Figure 1A and Figure 2A). 

The functional relationship between apoptosis and autophagy was further examined using the autophagy inhibitors, 3-methyladenine (3-MA) and chloroquine (Ch). Co-treatment with either inhibitor markedly enhanced the toxicity of all tested compounds (Figure 2B). These results clearly highlight the cytoprotective role of autophagy in the cellular response to drug-induced stress and suggest that combining the experimental hybrids with autophagy inhibitors may represent a promising strategy for potentiating anticancer efficacy.

### 2.3. The Involvement of Oxidative Stress in Metal-Tamoxifen Hybrids Action

**Figure 3:**
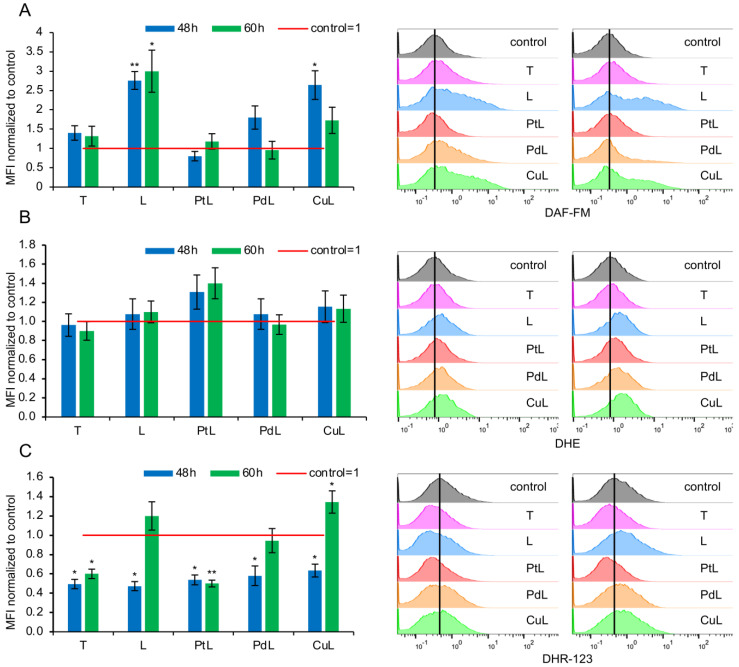
Experimental substances affected cellular redox status of 4T1 cells. Cells were exposed to an IC_50_ concentration of experimental compounds for 48 h and 60 h, and analyzed by flow cytometry: (**A**) NO detection (DAF-FM staining); (**B**) superoxide anion detection (DHE staining); (**C**) ROS/RNS detection (DHR-123 staining). * *p* > 0.05, ** *p* > 0.01, compared to control.

Chemotherapeutic agents containing a metal center often act by inducing excess production of reactive species, which subsequently damage intracellular structures and trigger cell death. Therefore, we examined cumulative ROS/RNS levels, as well as the current production of nitric oxide (NO) and superoxide anion (O_2_•^−^), after 48 and 60 h of treatment with the experimental compounds (Figure 3). 

The results revealed several unexpected cellular responses, especially considering the general assumption that apoptosis is usually closely associated with oxidative stress. Intracellular NO production was markedly elevated only in cells treated with the Cu–tamoxifen hybrid and the L alone, whereas Pd-based hybrid treatment induced only a transient and modest increase (Figure 3A). Superoxide production followed a similar but less pronounced pattern (Figure 3B). Surprisingly, the Pt-tamoxifen hybrid did not alter levels of either NO or O_2_•^−^, despite its strong cytotoxicity, reflected by its low IC_50_ value shown in Table 1. The assessment of cumulative ROS/RNS production—representing overall production of peroxynitrite (ONOO^−^), hydrogen peroxide (H_2_O_2_), hypochlorous acid (HOCl), and hydroxyl radicals generated through peroxynitrite decomposition—showed an unexpected suppression of total reactive species at 48 h across all treatments (Figure 3C). A moderate increase was observed only after 60 h in cultures exposed to the Cu–tamoxifen hybrid and the ligand alone. In contrast, the Pt–tamoxifen complex, similar to the active tamoxifen metabolite, displayed a persistent ROS-scavenging profile at both time points (48 and 60 h). Given that the tamoxifen metabolite does not rely on oxidative burst as its primary cytostatic mechanism, this observation was not unexpected. However, the general mechanism of action of metal-based chemotherapeutics typically involves enhanced production of reactive oxygen and nitrogen species. Thus, the moderate but detectable scavenging-like behavior of the Pt-based hybrid appeared contradictory to this paradigm. These findings prompted us to re-evaluate the biological activity of the Pt–tamoxifen hybrid and reassess its impact on 4T1 cell viability during the full 72 h incubation period, including an earlier time point than 48 h.

### 2.4. Different Dynamics of Intracellular Response to Pt-Based Tamoxifen Vector in 4T1 Cells and Metal Exchange Influence on Cell Repopulation

To demystify the unexpected effects of the Pt-tamoxifen hybrid, regarding the presence of Pt and its well-documented intracellular redox and proapoptotic performance and interpret them within a context of other experimental drugs with different metals, 4T1 cultures were exposed to the treatment for 24, 48, and 72 h-long periods, and all morphological changes were monitored by light microscopy.

**Figure 4:**
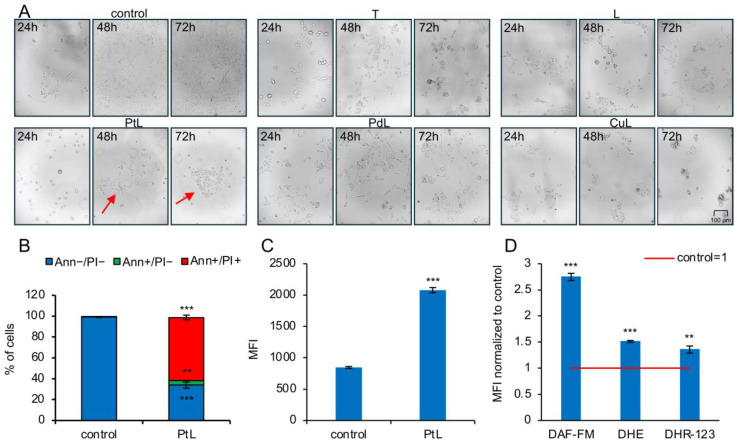
Pt-tamoxifen hybrid induced caspase-dependent apoptosis and oxidative stress in 4T1 cells as early as 24 h. (**A**) Cells treated with IC_50_ concentrations of experimental compounds for 24, 48, and 72 h analyzed by light microscopy (red arrows = cell repopulation); Cell were treated with IC_50_ concentration of Pt-tamoxifen hybrid for 24 h and analyzed by flow cytometry: (**B**) apoptosis detection (Annexin V-FITC/PI staining); (**C**) caspase activity (ApoStat staining), (**D**) detection of NO (DAF-FM staining), superoxide anion (DHE staining), and ROS/RNS (DHR-123 staining). ** *p* > 0.01, *** *p* > 0.001, compared to control.

As shown in Figure 4A, already after 24 h of exposure to the PtL, cells exhibited a predominantly rounded morphology and were largely detached from the culture dish surface—features characteristic for massive apoptotic cell death. In contrast, treatment with the CuL and particularly the Pd-hybrids resulted in less confluent cultures but with enlarged, flattened cells whose cytoplasm contained dense, dark granules, indicative of substantial drug uptake but without evident signs of apoptosis at this early time point.

After strong cytotoxic action within first 24 h of the treatment observed exclusively in PtL treated cultures with dominance of shrunk, rounded, almost detached cells, additional cultivation period led to microscopically visible cell recovery and colony formation, indicating cellular adaptation to drug-mediated cytotoxic activities (Figure 4A, red arrows).

In accordance with light microscopy, Annexin V-FITC/PI staining at the same time point confirmed a pronounced apoptotic response. Massive accumulation of cells in the late (double-positive) apoptotic stage (Figure 4B), followed by intensive caspase activation (Figure 4C) indicated rapid and efficient induction of apoptosis by the Pt-based hybrid within the first 24 h of incubation. To determine whether an early oxidative burst contributed to this effect, we performed DAF-FM, DHE, and DHR-123 staining at the 24 h time point. As shown in Figure 4D, the Pt-based tamoxifen hybrid triggered robust intracellular NO production, an increase in current superoxide anion content, and a pronounced accumulation of additional oxygen- and nitrogen-derived reactive species.

All together, these results showed that the Pt-tamoxifen hybrid exerts strong cytotoxic pressure within the first 24 h of TNBC 4T1 exposure, likely through a rapid and intense oxidative burst. However, further cultivation in the presence of this experimental drug led to an exhausted oxidative response. Cell repopulation clearly indicated the adaptation to the PtL cytotoxic action, a lower apoptotic cell death rate, and total caspase activity in comparison to other metal-tamoxifen hybrids as well as the ligand per se. Replacement of Pt with Pd, Cu, or even treatment with the ligand alone led to persistent antitumor activity without signs of cell repopulation.

### 2.5. Tamoxifen-Based Transition Metal Hybrids in Triple-Negative Breast Cancer Models In Vivo

**Figure 5:**
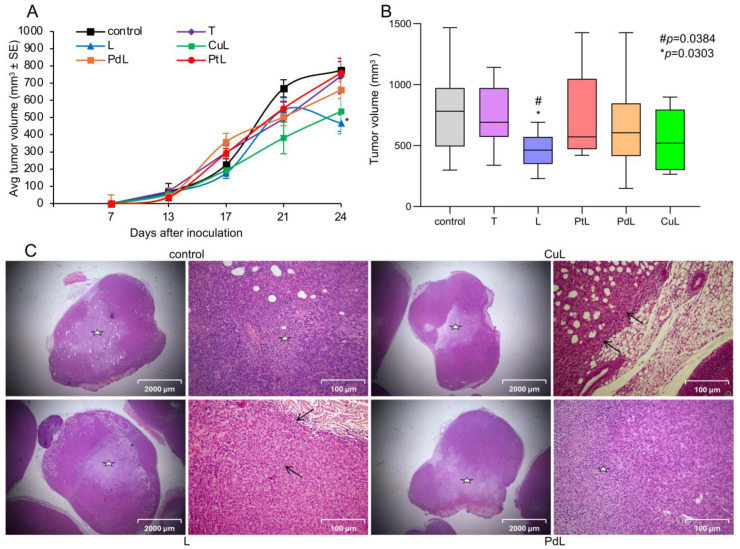
Treatment with ligand L results in a significant reduction in tumor volume: (**A**) Tumor growth curve obtained by measuring tumors in mice treated with experimental substances during 17 days. * *p* < 0.05 compared to control; (**B**) Volumes of tumors isolated from mice treated with experimental substances and measured at the moment of sacrification (24 days of inoculation)**.** Data are displayed as box-and-whisker plots representing tumor volume. The center line within each box represents the median, while the box boundaries indicate the 25th and 75th percentiles (interquartile range). The whiskers extend to the minimum and maximum values. Statistical significance was determined using Mann-Whitney U test. Significant differences are indicated as follows: * *p* < 0.05 compared to control, # *p* < 0.05 compared to T (**C**) Histopathological resections of tumors isolated from animals from the control, Cu-hybrid, L, and Pd-hybrid groups and stained with Hematoxylin and Eosin (H&E) (magnification: ×12.5 magnification (left) and ×200 (right)); white asterisks = areas of necrosis, black arrows = regions of lymphocytic infiltration.

To provide more relevant data about the efficacy of novel experimental hybrids, a syngeneic model of triple-negative breast cancer was employed. Tumors were induced by orthotopic inoculation of 4T1 cells into the fourth right mammary fat pad, and 5 days after inoculation, when tumors became palpable, treatment was started, simulating a therapeutic regimen. The schedule and doses were explained in detail in the Materials and Methods section. Tumors were measured every 4th day using a caliper, and tumor size was calculated. As shown in Figure 5A, tumor growth in animals treated with PtL as well as with tamoxifen, was only slightly and transiently inhibited, reaching values comparable to those of the control group by the end of the experiment. 

While the PdL exhibited a sustained antitumor effect with tumor growth continuously suppressed throughout the treatment period, notable tumor size reduction during the whole experiment, though not statistically significant, was achieved only in the case of Cu-tamoxifen-treated animals. The most intriguing tumor growth pattern was observed in animals treated with the tamoxifen ligand alone: although the effect emerged later, a pronounced reduction in tumor size became evident during the final week of the experiment, resulting in a significant tumor size decrease, compared with both the control group and all other tested compounds. Apart from the clear tendency of tumor size reduction obtained following the growth rate of tumors in animals treated with CuL, the tumor volume calculated from the dimensions of tumors collected at the end point of the experiment revealed a unique statistically significant reduction only in tamoxifen ligand-treated animals in comparison to the tamoxifen-treated group as well as the untreated control (Figure 5B). Concordantly, histopathological evaluation of tumor tissues isolated from animals in which treatment resulted in tumor growth reduction (PdL, CuL, L), revealed that pronounced immune cell infiltration, predominantly composed of lymphocytes, was observed exclusively in tumors isolated from tamoxifen ligand-treated mice (Figure 5C). These findings strongly support the claim that activation of tumor microenvironment–associated immune responses plays a key role in the delayed efficacy of the ligand treatment. In contrast, analysis of additional parameters—including the average number of mitoses per 2 mm^2^, the percentage of necrosis relative to total tumor area, and overall tumor morphology—showed no significant differences between treated groups and control animals (Table S1), with an exception of Cu-hybrid where necrotic area was moderately reduced in comparison to control samples (Figure 5C). The appearance of lymphocytic germ follicles in peritumoral tissue, as well as moderate tumor immune cell infiltration in CuL-treated animals, might be a sign of intensified antitumor immune response (Figure 5C) which can be beyond reduced tumor size through the following period of time. In parallel, tumor tissue architecture of PdL-treated animals didn’t differ significantly from controls, indicating that observed tumor growth reduction in this case cannot be explained by conventional histopathological metrics alone, and might be caused by other types of cell death. The treatments did not significantly affect body weight. However, visual observation revealed changes in appearance and behavior suggestive of potential toxicity exclusively in animals treated with Cu-hybrid. These included hunching, vocalization, piloerection, reduced activity, and decreased mobility.

Examination of kidney sections from control, non-treated tumor-bearing animals revealed the presence of proteinaceous material in the tubular lumen, passive hyperemia, and vascular dilatation, without inflammatory infiltrates (Figure S4). These findings are consistent with hemodynamic alterations rather than immune-mediated renal injury. Tumor–kidney cross-talk resulting in overt renal inflammation and fibrosis has primarily been described in advanced stages of disease. Therefore, the observed changes may reflect early systemic effects of tumor burden preceding the development of significant inflammatory and fibrotic remodeling. In contrast to the predominantly hemodynamic alterations observed in non-treated tumor-bearing animals, treated mice exhibited interstitial inflammatory infiltrates while in PdL-treated animals periglomerular sclerosis was detected additionally, suggesting progression toward structural renal remodeling.

Histopathological analysis of the liver in 4T1 tumor-bearing mice revealed a spectrum of changes reflecting systemic tumor effects and treatment-specific impacts (Figure S4). In control animals numerous foci of extramedullary hematopoiesis, accompanied by inflammatory cell infiltrates consistent with portal hepatitis, which are in concordance to previously described changes in this model [34], were demonstrated. Hepatocytes frequently exhibited binucleation and cytoplasmic vacuolization. Leukostasis was observed within hepatic vessels. The liver parenchyma showed marked cellularity, predominantly due to the extensive presence of inflammatory cells. Treatment with CuL and PdL induced additional features, including discrete periportal necrosis and scattered neutrophilic infiltrates, with neutrophils occasionally extending beyond the portal tracts. These findings suggest that the treatments contributed either to hepatocyte stress or enhanced immune-mediated responses superimposed on the systemic effects of the tumor injury.

## 3. Discussion

Reported “tamoxifen effects” in TNBC mainly arose from preclinical studies describing ER-independent or off-target mechanisms, e.g., mitochondrial stress, ROS induction, modulation of alternative receptors such as GPER1, and perturbation of sphingolipid metabolism and membrane-associated signaling nodes, with downstream consequences for cellular stress responses and survival pathways [19,35,36]. However, these findings have not translated into consistent clinical benefit, and tamoxifen is not considered an effective therapy for ER-negative TNBC patients.

Paradoxically, tumor heterogeneity and tamoxifen’s off-target activities keep this molecule relevant as a conceptual reference for designing hybrid compounds or discussing polypharmacology in TNBC therapy [37]. In this context, tamoxifen was mainly employed as a structural scaffold appropriate for delivering an additional cytotoxic moiety such as redox-active metal-based, or epigenetic component with well-defined antitumor potential against advanced forms of the disease rather than the drug per se. Tamoxifen has been combined with transition metals such as rhenium, ruthenium, titanium, osmium, and platinum. Scalcon et al. showed that two tamoxifen-derived pharmacophores, ‘hybrid’ metallo-drugs of Au(III) (AuTAML) and Cu(II) (CuTAML), synergized the anticancer activity of the metal center and the organic ligand against both ER-sensitive and triple-negative breast cancer cell lines. Ferrocenyl tamoxifen derivatives showed strong antiproliferative activity in TNBC cell lines (commonly MDA-MB-231) and, loaded into lipid nanocapsules, tested in a TNBC xenograft model significantly lowered tumor volume compared to the untreated group [5,6,38]. In addition, a well-known hybrid drug links a tamoxifen motif to an HDAC inhibitor, giving multi-target molecules that show enhanced activity against TNBC cells in vitro, again MDA-MB-231. Applying the well-known concept for anticancer drug design, a 2,2′-bipyridine-modified tamoxifen derivative was created by Schwarze, Kazimir, and colleagues and used for the design of hybrid molecules in which the tamoxifen served as a vector for molybdacarboranes [32], and later Pt(II), Pd(II), and Cu(II) [5,6], resulting in coordination complexes with significant antitumor potential against both, ER+ and TNBC cell line MDA-MB-231. This approach represents a polypharmacological strategy, where the tamoxifen scaffold acts not only as a drug but also as a lipophilic “carrier” inside hybrids, serving as a biologically validated anchor to generate additional antitumor mechanisms [39]. The final product should allow the simultaneous interplay between tamoxifen and metal center antitumor activity. The mechanisms of action of designed tamoxifen metal conjugates included combined cytostatic and cytotoxic effects, with inhibition of cell proliferation and induction of programmed cell death of types 1 (apoptosis) and 2 (autophagic cell death) [5,6]. The choice of metal was found to be essential for the redox response of the different breast cancer cells to the treatment, which varied from scavenging potential observed for tamoxifen carrier and complex with Pd, to oxidative burst observed for compounds based on Pt.

Recent reviews highlight that next-generation metallodrugs based on Ru, Au, Cu, and other transition metals show diverse anticancer activities and are considered promising alternatives to platinum drugs with broader mechanisms of action [20], distinct redox-mediated mechanisms compared to platinum drugs, and increased selectivity toward tumor tissue, alongside potentially reduced systemic toxicity [40]. Given the molecular heterogeneity, metabolic plasticity, and frequent DNA repair deficiencies characteristic of TNBC, metal-based therapeutics provide a mechanistically rational approach that extends beyond specific receptor dependency. However, the same features make them critically toxic for healthy tissues while tumor cells, far more adaptable than healthy cells due to their plasticity, become resistant to the cytotoxic effects of these drugs.

This study demonstrated that the hybrid molecules integrating 2,2′-bipyridine-modified tamoxifen derivative and metal subunit into one drug, exert antitumor activity in TNBC and with the same efficacy abrogate cell viability in cell lines belonging to other tumor types considered irrelevant for hormonal therapy. The previously observed sensitivity of human MDA-MB-231 cells to tamoxifen–metal derivatives has now gained a more general confirmation [5,6]. Moreover, a tendency toward a better response was noted in poorly differentiated cell lines, suggesting the possibility of targeting stemness and, consequently, more aggressive disease forms, i.e., potentially higher-grade tumors.

All tested tamoxifen–metal complexes exhibited cytotoxicity against tested cell lines in the low micromolar range, following the potency trend: CuL > PtL > PdL > L > T. The active tamoxifen metabolite used as an in vitro reference compound was at least 10 times less effective than newly designed hybrid drugs. Finally, subtle structural modification of tamoxifen with 2,2′-bipyridine (2,2′-bpy) chelating unit without metal incorporation resulted in remarkable enhancement of its ERα-independent cytotoxic action in comparison to the active tamoxifen metabolite, but was less efficient in comparison to hybrids in vitro, as expected.

Mechanistic analyses revealed distinct differences in the dynamics and magnitude of apoptosis induction among the tested compounds. The Pt-based tamoxifen hybrid induced a rapid and transient apoptotic response characterized by early caspase activation. However, caspase signaling subsequently declined, suggesting fast exhaustion of the apoptotic machinery. Surviving cells regained proliferative capacity, indicating the rapid emergence of drug-resistant clones. Intracellularly, this effect strongly correlated with an early and intense oxidative burst, characterized by predominant nitric oxide (NO) involvement within the first 24 h. This was followed by a subsequent decline in NO but also cumulative ROS/RNS production, suggesting a shift toward a scavenging profile compared to control, as reflected by the mean fluorescence intensity of DAF-FM and DHR-123 staining, respectively. Superoxide anion content showed a certain delay in comparison to NO, reaching the peak at the 48 h of the treatment, with a similar drop in an additional 12 h, manifested by DHE mean of fluorescence remarkably lower than in control cells. In contrast, Cu- and Pd-based hybrids, as well as the tamoxifen vector alone, triggered delayed but sustained caspase-dependent apoptotic response with a well-synchronized redox profile and dominance of NO and cumulative ROS/RNS production even after 60 h of incubation time. Light microscopy of 4T1 cultures clearly illustrated these divergent temporal patterns, with evident colony-forming units observed exclusively in cultures treated with the Pt-based hybrid. These findings are consistent with reports that substitution of platinum with other transition metals can significantly alter the mode of antitumor action and potentially mitigate resistance development. Finally, overall cytotoxicity was modulated by activation of autophagy, likely representing a cellular attempt to counteract drug-induced damage. Cytoprotective autophagy is a well-documented adaptive response that can attenuate the intracellular effects of anticancer agents and frequently contributes to therapeutic failure [18,20,21,32]. Although the compounds investigated in this study are assumed to share a broadly unified mechanism of action, the obtained data clearly demonstrate that the dynamics of intracellular events differ substantially depending on the metal coordinated to the ligand, and these differences are ultimately reflected in the treatment outcome in cell culture.

Translation of the in vitro findings into an in vivo context using a syngeneic orthotopic breast cancer model revealed a substantially altered therapeutic landscape once the tumor evolved within its native microenvironment with corresponding immune network involvement. In this setting, tumor progression is shaped not only by intrinsic cancer cell sensitivity but also by stromal interactions, vascularization, metabolic constraints, and both local and systemic immune surveillance [1,3,19,29,41]. Under these biologically relevant conditions, neither the Pt-based tamoxifen hybrid nor tamoxifen alone produced significant tumor growth inhibition throughout the experimental period. In concordance with in vitro observed rapid but transient apoptotic response and tumor cell renewal, the lack of in vivo efficacy observed with the Pt-hybrid may be additionally ascribed to multiple microenvironment-driven resistance mechanisms.

In contrast, the Cu–tamoxifen hybrid induced sustained but statistically insignificant tumor growth suppression, indicating a more robust and durable antitumor mechanism in vivo. Given the known redox activity of copper complexes, it is plausible that persistent oxidative stress and prolonged apoptotic signaling limited tumor recovery and clonal adaptation within the microenvironment [42]. Such dynamics in the induction of cell death and the pronounced cytotoxic potential observed in vitro can indicate the immunogenicity of the treatment within the tumor microenvironment. Appearance of lymphocytic germ follicles in peritumoral tissue, and immune cell infiltration visualized on tumor sections, strongly supported this hypothesis as a valuable sign of treatment-induced immune activation. Metal replacement by Pd decreased this capacity and underlined different modes of Pd action which are under investigation and refer to different types of cell death or senescence induction. The discrepancy between tumor growth reduction and the preservation of tissue architecture is a hallmark of non-immunogenic, regulated cell death [43,44]. Our in vitro data confirms that the Pd-complex triggers cytoprotective autophagy and caspase-dependent apoptosis. We may assume that autophagy delays the transition to cell death, ensuring that, when the cell finally undergoes apoptosis, the process is done without the release of pro-inflammatory DAMPs (Damage-Associated Molecular Patterns) [44,45]. This secures apoptosis as a gradual, morphologically “silent” process, unlike necrosis, which involves membrane rupture and the massive release of intracellular constituents [43]. Also, palladium complexes have been reported to exert anti-angiogenic and anti-invasive effects, potentially leading to a slow starvation of the tumor [46]. Low-level nutrient deprivation typically results in steady, individual cell loss through apoptosis rather than mass tissue death through necrosis [47]. Furthermore, the high affinity of palladium, similarly to Cu, for thiol groups (such as glutathione and thioredoxin reductase) may induce ferroptotic or cuproptotic-like metabolic stress [43,45,46,48]. This biochemical failure can lead to cell death through internal systems failure without disrupting the macroscopic tissue architecture [44,47]. However, restricted immunological visibility might be responsible for limited tumor reducing potential of Pd-based hybrids. The most striking and conceptually important observation was obtained with the subtly structurally modified tamoxifen used as a ligand when it was applied alone. Although its early antitumor effect was modest, a pronounced reduction in tumor mass emerged during the final days of the experiment. Histological examination revealed extensive immune cell infiltration with dominance of lymphocytes, exclusively in tumors isolated from ligand-treated animals. This pattern strongly indicates that the ligand did not primarily act through direct cytotoxicity, but rather through modulation of the tumor microenvironment and restoration of efficient immune-mediated tumor control. It is well documented that in certain cancer models, the regression of tumor mass was not just the consequence of drug’s direct toxicity, but was mediated by infiltration of inflammatory immune cells in response to intratumoral changes provoked by the treatment. Without this immunogenicity, the long-term efficacy of the applied therapy was significantly reduced [49,50]. Because the immune cells must physically migrate and expand within the tumor stroma in response to immunogenic stimuli, the measurable decrease in tumor burden often lags behind the start of treatment [51,52]. Overall, such delayed tumor regression accompanied by immune infiltration is compatible with mechanisms involving immunogenic stress, alteration of cytokine signaling, or reprogramming of suppressive components within the tumor microenvironment. Furthermore, therapy-induced senescence (TIS) like that observed upon the treatment with ligand in cell culture visualised by the presence of large nuclei and flattened cell morphology, in vivo may contribute to the pronounced lymphocytic infiltration observed in treated TNBC tumors by establishing a pro-inflammatory, immune-recruiting secretory program (SASP). In an orthotopic 4T1 TNBC model, induction of tumor senescence together with SASP reprogramming increased intratumoral CD8^+^ T cells and NK cells, enhanced cytotoxic activity (e.g., degranulation/granzyme B), and shifted the local cytokine milieu toward a more immunostimulatory state, consistent with strengthened local immune surveillance [53]. Collectively, these findings suggest that while metal-based hybrids predominantly exert direct cytotoxic pressure which leads to immunogenic death in Cu-based drugs, the tamoxifen ligand alone may function as an immunomodulatory trigger capable of reactivating endogenous antitumor immunity.

This distinction highlights an important therapeutic concept: durable tumor control in vivo may depend less on the magnitude of acute cytotoxicity and more on the ability to reshape the tumor–immune equilibrium. In addition to histopathological analyses, which indicated moderate structural deficiencies in liver and kidneys in animals exposed to the Cu-, Pd-, and tamoxifen ligand alone, continuous clinical observation of the animals indicated clear signs of systemic toxicity associated only with the Cu–tamoxifen hybrid. This adverse effect was manifested by visible signs of physiological distress, suggesting that despite its antitumor efficacy, the therapeutic window of the Cu-based complex may be limited.

Design of hybrid tamoxifen–metal conjugates was believed to be a promising strategy for the treatment of TNBC, a malignancy characterized by the absence of actionable molecular targets and frequent therapeutic resistance. The modular incorporation of bioactive metal centers enables diversification of mechanisms of action resulting in improved drug features such as prolonged efficacy and lack or delay in resistance establishment in vitro. Since the promising in vitro activity was not properly maintained in the complex setting of the tumor microenvironment and the intact organism, the translational relevance of these findings remains uncertain, but highly informative in terms of further interventions that can amplify their in vivo performance like additional chemical interventions and/or targeted delivery using nanotechnology.

However, practically and fundamentally valuable outcome of the study is the discovery that delicate structural modification of tamoxifen by 2,2′-bipyridine (2,2′-bpy) chelating unit addition can substantially enhance its therapeutic performance without the necessary incorporation of a metal subunit. Notably, this improvement appears to arise not only from direct cytotoxic effects on tumor cells but also from indirect modulation of the tumor microenvironment, potentially leading to improved antitumor efficacy with reduced systemic toxicity. Having in mind that on the list of tamoxifen off-targets, cannabidiol receptors take an important place, together with the fact that the same receptors are expressed in multiple cell compartments within the tumor microenvironment, including TNBC cells as well as immune cells [15], one of the directions that should be addressed in the future is whether modified tamoxifen, as well as its hybrid metal-based variants, cooperate with the aforementioned receptors and how these interactions can influence the tumor microenvironment with an accent on cancer-immune-stromal cells crosstalk.

## 4. Materials and Methods

### 4.1. Reagents and Cell Culture

The following reagents were purchased from Sigma (St. Louis, MO, USA): dimethyl sulfoxide (DMSO), crystal violet (CV), phosphate-buffered saline (PBS), propidium iodide (PI), carboxyfluorescein diacetate succinimidyl ester (CFSE), and acridine orange (AO). Paraformaldehyde (PFA) was obtained from SERVA Electrophoresis GmbH (Heidelberg, Germany), while 3-(4,5-dimethylthiazol-2-yl)-2,5-diphenyltetrazolium bromide (MTT) was sourced from AppliChem (Darmstadt, Germany). Cell culture medium (HEPES-buffered RPMI-1640) and fetal calf serum (FCS) were provided by Capricorn Scientific GmbH (Ebsdorfergrund, Germany). Penicillin-streptomycin solution was acquired from Biological Industries (Cromwell, CT, USA). Annexin V-FITC (AnnV) was purchased from BD Pharmingen (San Diego, CA, USA), and the ApoStat probe was obtained from R&D Systems (Minneapolis, MN, USA). Dihydrorhodamine 123 (DHR 123) and dihydroethidium (DHE) were sourced from Thermo Fisher Scientific (Waltham, MA, USA). The manufacturer of diaminofluorescein (DAF)-FM diacetate is Enzo Life Sciences (Farmingdale, NY, USA). Additional supplies were procured from Bio-Optica Milano S.p.A, Milano, Italy.

The murine cell lines used in this study—4T1 (triple-negative breast cancer), B16, and B16F10 (melanoma)—were obtained from the American Type Culture Collection (ATCC, Manassas, VA, USA). Cells were maintained in HEPES-buffered RPMI-1640 medium supplemented with 10% heat-inactivated FCS, 2 mM L-glutamine, 0.01% sodium pyruvate, 100 units/mL penicillin, and 100 µg/mL streptomycin. Cultures were incubated at 37 °C in a humidified environment with 5% CO_2_.

### 4.2. Synthesis and Characterization of Complexes

The synthetic procedures, compound stability, and detailed structural characterization of the reported complexes (NMR, X-ray diffraction (XRD), UV–Vis, and IR spectroscopy) have been described comprehensively in our previous publications [5,6].

### 4.3. Preparation of Compounds and Experimental Design

Stock solutions of tamoxifen derivatives were prepared in DMSO at a concentration of 20 mM, 4,4′-dihydroxytamoxifen at a concentration of 20 mM, and stored at −20 °C. Working concentrations were freshly prepared in culture media prior to each experiment. For viability assays, 4T1 (3 × 10^3^ cells/well), B16 (4 × 10^3^ cells/well), and B16F10 (3 × 10^3^ cells/well) were seeded in 96-well plates. For flow cytometry and light microscopy, 4T1 cells were seeded at a density of 1 × 10^5^ cells per well in 6-well plates.

### 4.4. Viability Assays

Cell viability was evaluated 72 h post-exposure to varying doses of T, L, PtL, PdL, CuL using MTT and CV assays.

MTT Assay: Following treatment, the supernatant was removed, and cells were incubated with 0.5 mg/mL MTT solution for approximately 30 min at 37 °C. The resulting formazan crystals were dissolved in DMSO.

CV Assay: Cells were fixed with 4% PFA for 10 min at room temperature, then stained with 0.02% CV solution for 15 min. After washing with tap water and air-drying, the dye was solubilized in 33% acetic acid.

For both assays, absorbance was measured at 540 nm and 670 nm using an automated microplate reader. Results are expressed as a percentage relative to untreated controls (which represent 100% viability). IC_50_ values were determined using a four-parameter logistic function and represent the mean of three independent experiments.

### 4.5. Microscopy

Light microscopy: In order to detect and identify morphological and intracellular changes at the microscopic level, 4T1 cells were seeded and treated in the absence or presence of the IC_50_ values of the tested compounds. After 24, 48 and 72 h, cells were observed without prior staining.

Fluorescence microscopy: 4T1 cells were exposed to the IC_50_ concentrations of tested compounds for a duration of 72 h. Following the incubation period, the cells were fixed using a solution of 4% paraformaldehyde (PFA) for 15 min at room temperature and subsequently stained by propidium iodide (PI 50 μg/mL, 0.1% Triton X-100, 0.1 mM EDTA pH 8.0, and RNase 85 μg/mL in phosphate-buffered saline (PBS)) for a duration of 2 min.

The prepared cells were digitally photographed on a ZOE Fluorescent Cell Imager (Bio-Rad Laboratories, Hercules, CA, USA). Software used for image editing was ImageJ 1.54g.

### 4.6. Flow Cytometric Analyses

All flow cytometric data were acquired using a CytoFLEX^®^ Flow Cytometer (Beckman Coulter, Indianapolis, IN, USA) while the analysis of the obtained results was performed using the FlowJo™ software program (Version 10).

Proliferation (CFSE): 4T1 cells were labeled with 1 µM CFSE for 10 min at 37 °C. After washing, cells were seeded, treated with IC_50_ doses of experimental substances for 72 h, and subsequently harvested in PBS for further analysis.

Apoptosis (Ann V/PI, ApoStat) and Autophagy (AO): Following treatment with IC_50_ doses T, L, PtL, PdL, and CuL for 24, 48, and 60 h, cells were harvested and washed. Apoptosis was assessed via Ann V/PI dual staining (PI at 15 µg/mL) for 15 min at room temperature. Caspase activity was detected using the ApoStat pan-caspase inhibitor (30-min incubation at 37 °C). Autophagic vacuoles were identified by staining with 10 µM acridine orange for 15 min at 37 °C.

ROS/RNS production (DHR-123): Reactive species generation was measured using 1 µM DHR 123. Cells were pre-stained with DHR 123 for 20 min at 37 °C before treatment with IC_50_ dose of the tested agents for 24, 48, and 60 h. Post-treatment, cells were washed, trypsinized, and analyzed.

NO production (DAF-FM diacetate): Cells were pre-incubated with a 5 µM working solution of DAF-FM diacetate in phenol red-free RPMI 1640 medium (“white medium”) supplemented with 10% FCS for 1 h at 37 °C. Following staining, cells were washed twice with PBS and further incubated in FCS-free white medium for 15 min at 37 °C to facilitate complete de-esterification of intracellular diacetates. After washing and detachment via trypsinization, cells were harvested in white medium with 10% FCS and centrifuged at 750 *g* for 3 min. The resulting pellets were washed, resuspended in 1 mL of PBS, and maintained on ice until flow cytometric analysis.

Superoxide Anion detection (DHE): Following treatment, the culture medium was discarded, and cells were washed with PBS before being detached by trypsinization. The reaction was neutralized with cell culture medium containing 10% FCS, and cells were harvested into FACS tubes and centrifuged at 750× *g* for 3 min. Pellets were then resuspended in 0.1 mL of a 10 µM working DHE solution and incubated in the dark at room temperature for 30–45 min. After staining, cells were washed twice with PBS via centrifugation (750× *g* for 3 min), resuspended in 1 mL of PBS, and kept on ice for immediate analysis.

### 4.7. In Vivo Study

Animals used in this study were female inbred BALB/c mice, 8 weeks old, from the Institute for Biological Research “Siniša Stanković”, National Institute of Republic of Serbia (IBISS). Animals were kept in standard pathogen-free laboratory conditions, with ad libitum regime of food and water intake. The study protocol as well as the handling of animals were in concordance with the national regulations established by the Law on Animal Welfare of the Republic of Serbia (Official Gazette of the Republic of Serbia No. 41/2009) and European Ethical Normative (Directive 2010/63/EU) on the protection of animals used for experimental and other scientific purposes. The national licensing committee at the Department of Animal Welfare, Veterinary Directorate, Ministry of Agriculture, Forestry and Water Management of Republic of Serbia granted approval for the experimental protocols (permission No. 323-07-05657/2022-05).

Female BALB/c mice were orthotopically inoculated with 4T1 cells (2 × 10^4^ cells/50 µL PBS) in the fat pad region of the fourth mammary gland. The treatment of animals started on the fifth day following cell implantation, when tumors became palpable. The animals were randomly divided into six groups, with the number of mice per group being 8–10. The administration regime consisted of five consecutively i.p. applications of the appropriate agent, after which two-day treatment-free period was applied. Used doses of each substance were: Tamoxifen 10 mg/kg, PtL 18.4 mg/kg, PdL 16 mg/kg, CuL 5 mg/kg, and L 11.4 mg/kg, all administered in 2% DMSO/PBS. The doses of experimental drugs were established on an equimolar basis relative to tamoxifen, whose 10 mg/kg dose is well documented as a therapeutic concentration in murine breast cancer studies [54,55], except for the Cu-based compound, whose dose was determined based on the results of a preliminary pilot experiment and corresponded to other compounds according to its dimeric structure. Mice in the control group were receiving 2% DMSO/PBS as vehicle. Tumor growth was monitored every third day, and mice were sacrificed on the 24th day after cell implementation. Tumor tissues, livers and kidneys were collected from all groups for further histological analysis. Upon extraction, tumors’ three dimensions were measured and the tumor volume (mm^3^) was calculated according to formula: length × width^2^ × 0.52. During the whole experiment, changes in the animals’ body weight, the overall state as well as behavioral changes were observed.

Formalin-Fixed Paraffin-Embedded (FFPE) Tissue Preparation and Hematoxylin and Eosin (H&E) Staining Protocol

Following sacrifice, the tumors, livers, and kidneys of the experimental animals were subjected to gross examination and subsequently fixed in 10% neutral buffered formalin (NBF) at a fixative-to-tissue ratio of 10:1 for 24 h. Tissues were then sectioned along their largest axis and returned to fixative for an additional 24-h period to ensure optimal preservation. Tissue processing was performed using an automated tissue processor (LOGOS ONE, Milestone SRL, Bergamo, Italy), and paraffin embedding was carried out with an embedding station (Tissue-Tek TEC 5, SAKURA, Los Angeles, CA, USA). Paraffin blocks were sectioned at 4 μm thickness using a microtome (RM2245, LEICA, Nussloch, Germany), and the sections were mounted onto glass slides. Slides were incubated at 60 °C for 15 min, deparaffinized in xylene, and rehydrated through a graded ethanol series.

Hematoxylin and eosin (H&E) staining was performed on an automated stainer (SS-30H, MYREVA, Tarragona, Spain). Slides were stained with hematoxylin for 5 min, rinsed in water, differentiated in acid alcohol, and blued using either tap water or a blueing reagent. Counterstaining with eosin was performed for 2 min, followed by dehydration in ethanol, clearing in xylene, and coverslipping.

Histopathological evaluation was conducted using an Olympus BX43 light microscope (OLYMPUS EUROPA HOLDING GMBH, Hamburg, Germany).

### 4.8. Statistical Analysis

Experimental data are presented as the mean +/− standard deviation (SD) from at least three independent biological replicates. Differences between treatments in the in vitro studies were analyzed using Student’s *t*-test, with *p* < 0.05 considered statistically significant. The non-parametric Mann–Whitney U test was used for statistical analysis in the in vivo study. Data analysis software Statistica (version 12), was used for the statistical calculation.

## 5. Conclusions

Beyond its primary role as a selective estrogen receptor antagonist, tamoxifen exhibits a pronounced antitumor potential that is not exclusively dependent on its pivotal role. Numerous off-target mechanisms with its favorable pharmacokinetic and biochemical properties that enable its efficient uptake and accumulation within malignant cells have positioned tamoxifen as a suitable molecular carrier for the delivery of cytotoxic agents, as well as a versatile platform for the design of hybrid compounds [5,6,12,32,35]. Tamoxifen derivative 4-[1,1-bis(4-methoxyphenyl)but-1-en-2-yl]-2,2′-bipyridine was used for the design of hybrid molecules containing Pt-, Pd-, or Cu-based metal cores as active subunits [5,6,32]. Newly designed conjugates as well as tamoxifen vectors exerted high cytotoxic potential in vitro with 10 times amplified tumoricidal activity of metal-based hybrids in comparison to active tamoxifen metabolites. This study reveals the influence of metal exchange not principally to intensity, but to the dynamic and duration of programmed cell death, resulting in a switch from rapid and transient apoptosis induction observed upon Pt-based drugs to a delayed, slow, but persistent process, leading to permanent cytotoxicity during all tested periods in vitro with Pd- and Cu-counterparts. This dynamic was in strict correlation with the kinetics of intracellular NO content as well as the accumulation of total ROS and RNS species. The extent to which cytotoxicity is not decisive in assessing the efficacy of a drug is best demonstrated by the results presented here, where evaluation in an in vivo model showed that the tamoxifen vector itself, with significantly lower activity in vitro compared to hybrids, has the strongest potential in tumor reduction, accompanied by intensive accumulation of immune cell infiltrates, preferentially lymphocytes. This is consistent with the growing body of evidence about the compensatory response of advanced tumors to aggressive therapy, as well as with the fact that reanimation of the antitumor immune response is one of the most long-term effective approaches in the cancer treatment. Finally, these findings underscore how minor chemical refinements within a vector–metal framework can profoundly influence biological outcome, therapeutic selectivity, and the balance between efficacy and tolerability. The results presented in this study, together with our previous findings, indicate that the modified tamoxifen vector displays substantially improved properties compared with the parent drug, making it an attractive candidate for further investigation and potential therapeutic application in both ERα-positive breast cancer and aggressive triple-negative breast cancer.

## Figures and Tables

**Table 1:** IC_50_ values (μM) for T, L, PtL, PdL, and CuL on different cancer cell lines obtained by MTT and CV assays after 72 h of incubation shown as mean of three independent experiments ± SD.

Compound	Assay	4T1	B16	B16F10
T	MTT	22.95 ± 1.63	24.25 ± 0.78	28.35 ± 2.33
	CV	32.70 ± 0.57	27.55 ± 0.21	47.10 ± 0.71
L	MTT	3.60 ± 0.00	4.97 ± 0.12	4.95 ± 0.07
	CV	4.58 ± 0.17	5.47 ± 0.06	6.25 ± 0.21
PtL	MTT	1.80 ± 0.28	2.40 ± 0.01	1.85 ± 0.21
	CV	2.45 ± 0.07	2.50 ± 0.14	2.45 ± 0.35
PdL	MTT	3.55 ± 0.07	6.55 ± 0.50	4.97 ± 0.31
	CV	4.23 ± 0.35	6.40 ± 0.57	5.60 ± 0.28
CuL	MTT	0.85 ± 0.21	1.20 ± 0.10	0.93 ± 0.06
	CV	1.00 ± 0.14	1.27 ± 0.06	1.17 ± 0.06

## Data Availability

The data presented in this study are available in the article or Supplementary Materials.

## Supplementary Materials

The following supporting information can be downloaded at: https://www.mdpi.com/article/10.3390/molecules31091376/s1, Table S1: Histological assessment of the mitotic rate and necrosis percentage of tumors isolated from animals treated with L, PdL, and CuL obtained and calculated by light microscopy; Figure S1: All experimental compounds decrease cell viability in dose-dependent manner. The viability of 4T1, B16, and B16F10 cells treated for 72 h with wide range of concentrations of experimental compounds was determined by MTT (left panel) and CV (right panel) assays. Viability was expressed as a percentage of the absorbance value of control cells that was arbitrary assigned a viability value of 100%. Data shown represent SV ± SD of one representative of 3 independent experiments. * *p* > 0.05 compared to control; Figure S2: L and Pd-based hybrid induce moderate cell proliferation arrest. 4T1 cells were treated with IC_50_ concentrations of experimental compounds in the presence of the CFSE dye for 72 h and analyzed by flow cytometry. * *p* > 0.05 compared to control; Figure S3: Ligand influences the enlargement of the cells. 4T1 were treated with IC_50_ concentrations of experimental agents for 72 h, digitally photographed by ZOE fluorescent cell imager and analyzed by photo analysis software. * *p* > 0.05 compared to control; Figure S4: Histopathological resections of kidney (upper panels) and liver (lower panels) tissues isolated from animals from the control (A,E), Cu-hybrid (B,F), L (C,G), and Pd-hybrid (D,H) groups and stained with Hematoxylin and Eosin (H&E) (×200 magnification); white asterisks = areas of necrosis, black arrows = protein casts within renal tubules, white arrow = inflammatory infiltrate in the kidney, broad white arrows = periglomerular fibrosis, thin white arrows = focus of extramedullary hematopoiesis in the liver, curved arrows = focal neutrophilic infiltration.

## Abbreviations

The following abbreviations are used in this manuscript:

T	4,4′-dihydroxytamoxifen
L	4-[1,1-bis(4-methoxyphenyl)but-1-en-2-yl]-2,2′-bipyridine
PtL	[PtCl_2_(L-κ^2^*N*,*N*′)]
PdL	[PdCl_2_(L-κ^2^*N*,*N*′)]
CuL	[CuCl(μ-Cl)(L-κ^2^*N*,*N*′)]_2_
TNBC	Triple-negative breast cancer
DAF-FM	4-amino-5-methylamino-2′,7′-difluorofluorescein diacetate
DHE	Dihydroethidium
DHR-123	Dihydrorhodamine 123
HER2	Human epidermal growth factor receptor 2
ER	Estrogen receptor
PR	Progesterone receptor
BC	Breast cancer
SERM	Selective estrogen receptor modulator
ERα	Estrogen receptor alpha
ERE	Estrogen response elements
LBD	Ligand-binding domain
GPER1	G protein-coupled estrogen receptor 1
HIF-1A	Hypoxia inducible factor 1 alpha
CB1-R	Cannabinoid receptor 1
CB2-R	Cannabinoid receptor 2
PKC	Protein kinase C
NRF2	Nuclear factor erythroid 2-related factor 2
CSC	Cancer stem cells
PI3K	Phosphatidylinositol 3-kinases
AKT	Protein Kinase B
mTOR	Mechanistic target of rapamycin
MAPK	Mitogen-activated protein kinases
ERK	Extracellular signal-regulated kinases
ROS/RNS	Reactive oxygen and nitrogen species
CFSE	Carboxyfluorescein diacetate succinimidyl ester
PI	Propidium iodide
Ch	Chloroquine
3-MA	3-methyladenine
NO	Nitric oxide
SERD	Selective estrogen receptor degrader
CDK4/6	Cyclin-dependent kinases 4/6
DAMP	Damage-Associated Molecular Patterns
TIS	Therapy-induced senescence
SASP	Immune-recruiting secretory program

## References

[1] Vojtek M., Marques M.P.M., Ferreira I.M.P.L.V.O., Mota-Filipe H., Diniz C. (2019). Anticancer Activity of Palladium-Based Complexes against Triple-Negative Breast Cancer. Drug Discov. Today.

[2] Waks A.G., Winer E.P. (2019). Breast Cancer Treatment: A Review. JAMA.

[3] Mishra A., Srivastava A., Pateriya A., Tomar M.S., Mishra A.K., Shrivastava A. (2021). Metabolic Reprograming Confers Tamoxifen Resistance in Breast Cancer. Chem.-Biol. Interact..

[4] Tsang J.Y.S., Tse G.M. (2019). Molecular Classification of Breast Cancer. Adv Anat Pathol..

[5] Kazimir A., Schwarze B., Lönnecke P., Jelača S., Mijatović S., Maksimović-Ivanić D., Hey-Hawkins E. (2023). Metallodrugs against Breast Cancer: Combining the Tamoxifen Vector with Platinum(II) and Palladium(II) Complexes. Pharmaceutics.

[6] Kazimir A., Schwarze B., Lönnecke P., Jelača S., Mijatović S., Maksimović-Ivanić D., Hey-Hawkins E. (2023). Exploring the Potential of Tamoxifen-Based Copper(II) Dichloride in Breast Cancer Therapy. RSC Med. Chem..

[7] Kiyotani K., Mushiroda T., Nakamura Y., Zembutsu H. (2012). Pharmacogenomics of Tamoxifen: Roles of Drug Metabolizing Enzymes and Transporters. Drug Metab. Pharmacokinet..

[8] Azzam H.N., El-Derany M.O., Wahdan S.A., Faheim R.M., Helal G.K., El-Demerdash E. (2023). The Role of Mitochondrial/Metabolic Axis in Development of Tamoxifen Resistance in Breast Cancer. Hum. Cell.

[9] Shen J., He Y., Li S., Chen H. (2024). Crosstalk of Methylation and Tamoxifen in Breast Cancer (Review). Mol. Med. Rep..

[10] Drăgănescu M., Carmocan C. (2017). Hormone Therapy in Breast Cancer. Chirurgia.

[11] Ghosh S. (2019). Cisplatin: The First Metal Based Anticancer Drug. Bioorganic Chem..

[12] Ahmed N.S., Samec M., Liskova A., Kubatka P., Saso L. (2021). Tamoxifen and Oxidative Stress: An Overlooked Connection. Discov. Oncol..

[13] Wang Q., Jiang J., Ying G., Xie X.-Q., Zhang X., Xu W., Zhang X., Song E., Bu H., Ping Y.-F. (2018). Tamoxifen Enhances Stemness and Promotes Metastasis of ERα36+ Breast Cancer by Upregulating ALDH1A1 in Cancer Cells. Cell Res..

[14] Cortes E., Lachowski D., Robinson B., Sarper M., Teppo J.S., Thorpe S.D., Lieberthal T.J., Iwamoto K., Lee D.A., Okada-Hatakeyama M. (2019). Tamoxifen Mechanically Reprograms the Tumor Microenvironment via HIF -1A and Reduces Cancer Cell Survival. EMBO Rep..

[15] Kisková T., Mungenast F., Suváková M., Jäger W., Thalhammer T. (2019). Future Aspects for Cannabinoids in Breast Cancer Therapy. Int. J. Mol. Sci..

[16] Sfogliarini C., Pepe G., Dolce A., Della Torre S., Cesta M.C., Allegretti M., Locati M., Vegeto E. (2022). Tamoxifen Twists Again: On and Off-Targets in Macrophages and Infections. Front. Pharmacol..

[17] Abdel-Hafiz H. (2017). Epigenetic Mechanisms of Tamoxifen Resistance in Luminal Breast Cancer. Diseases.

[18] Yao J., Deng K., Huang J., Zeng R., Zuo J. (2020). Progress in the Understanding of the Mechanism of Tamoxifen Resistance in Breast Cancer. Front. Pharmacol..

[19] Osborne C.K., Schiff R. (2011). Mechanisms of Endocrine Resistance in Breast Cancer. Annu. Rev. Med..

[20] Adhikari S., Nath P., Das A., Datta A., Baildya N., Duttaroy A.K., Pathak S. (2024). A Review on Metal Complexes and Its Anti-Cancer Activities: Recent Updates from in Vivo Studies. Biomed. Pharmacother..

[21] Lucaciu R.L., Hangan A.C., Sevastre B., Oprean L.S. (2022). Metallo-Drugs in Cancer Therapy: Past, Present and Future. Molecules.

[22] Riccardi C., Piccolo M. (2023). Metal-Based Complexes in Cancer. Int. J. Mol. Sci..

[23] Lin C., Cui J., Peng Z., Qian K., Wu R., Cheng Y., Yin W. (2022). Efficacy of Platinum-Based and Non-Platinum-Based Drugs on Triple-Negative Breast Cancer: Meta-Analysis. Eur. J. Med. Res..

[24] Dasari S., Bernard Tchounwou P. (2014). Cisplatin in Cancer Therapy: Molecular Mechanisms of Action. Eur. J. Pharmacol..

[25] Wang D., Lippard S.J. (2005). Cellular Processing of Platinum Anticancer Drugs. Nat. Rev. Drug Discov..

[26] Wimmer F.L., Wimmer S., Castan P., Cros S., Johnson N., Colacio-Rodrigez E. (1989). The Antitumor Activity of Some Palladium(II) Complexes with Chelating Ligands. Anticancer Res..

[27] Zhao G., Lin H., Ping Y., Sun H., Zhu S., Xuncheng S., Chen Y. (1999). Ethylenediamine-Palladium(II) Complexes with Pyridine and Its Derivatives: Synthesis, Molecular Structure and Initial Antitumor Studies. J. Inorg. Biochem..

[28] Shan D., Song J., Ren Y., Zhang Y., Ba Y., Luo P., Cheng Q., Xu H., Weng S., Zuo A. (2025). Copper in Cancer: Friend or Foe? Metabolism, Dysregulation, and Therapeutic Opportunities. Cancer Commun..

[29] Tang X., Yan Z., Miao Y., Ha W., Li Z., Yang L., Mi D. (2023). Copper in Cancer: From Limiting Nutrient to Therapeutic Target. Front. Oncol..

[30] Richard M., Hamels D., Pigeon P., Top S., Dansette P.M., Lee H.Z.S., Vessières A., Mansuy D., Jaouen G. (2015). Oxidative Metabolism of Ferrocene Analogues of Tamoxifen: Characterization and Antiproliferative Activities of the Metabolites. ChemMedChem.

[31] Top S., Kaloun E.B., Vessières A., Leclercq G., Laïos I., Ourevitch M., Deuschel C., McGlinchey M.J., Jaouen G. (2003). Tamoxifen Derivatives for Delivery of the Antitumoral (DACH)Pt Group: Selective Synthesis by McMurry Coupling, and Biochemical Behaviour. ChemBioChem.

[32] Schwarze B., Jelača S., Welcke L., Maksimović-Ivanić D., Mijatović S., Hey-Hawkins E. (2019). 2,2′-Bipyridine-Modified Tamoxifen: A Versatile Vector for Molybdacarboranes. ChemMedChem.

[33] Marzagalli M., Montagnani Marelli M., Casati L., Fontana F., Moretti R.M., Limonta P. (2016). Estrogen Receptor β in Melanoma: From Molecular Insights to Potential Clinical Utility. Front. Endocrinol..

[34] duPre’ S.A., Hunter K.W. (2007). Murine Mammary Carcinoma 4T1 Induces a Leukemoid Reaction with Splenomegaly: Association with Tumor-Derived Growth Factors. Exp. Mol. Pathol..

[35] Morad S.A.F., Cabot M.C. (2015). Tamoxifen Regulation of Sphingolipid Metabolism—Therapeutic Implications. Biochim. Biophys. Acta (BBA)—Mol. Cell Biol. Lipids.

[36] Filardo E.J., Quinn J.A., Bland K.I., Frackelton A.R. (2000). Estrogen-Induced Activation of Erk-1 and Erk-2 Requires the G Protein-Coupled Receptor Homolog, GPR30, and Occurs via Trans-Activation of the Epidermal Growth Factor Receptor through Release of HB-EGF. Mol. Endocrinol..

[37] Kent Osborne C. (1993). Mechanisms for Tamoxifen Resistance in Breast Cancer: Possible Role of Tamoxifen Metabolism. J. Steroid Biochem. Mol. Biol..

[38] Lainé A.-L., Adriaenssens E., Vessières A., Jaouen G., Corbet C., Desruelles E., Pigeon P., Toillon R.-A., Passirani C. (2013). The in Vivo Performance of Ferrocenyl Tamoxifen Lipid Nanocapsules in Xenografted Triple Negative Breast Cancer. Biomaterials.

[39] Shagufta, Ahmad I. (2018). Tamoxifen a Pioneering Drug: An Update on the Therapeutic Potential of Tamoxifen Derivatives. Eur. J. Med. Chem..

[40] Alsabeelah N.F. (2026). Exploring the Therapeutic Potential of Metal Complexes in Glioblastoma Treatment: A Focus on Brain Tumor Targeting Strategies. Rev. Inorg. Chem..

[41] Sari D., Gozuacik D., Akkoc Y. (2024). Role of Autophagy in Cancer-Associated Fibroblast Activation, Signaling and Metabolic Reprograming. Front. Cell Dev. Biol..

[42] Moreno-Narváez M.E., González-Sebastián L., Colorado-Peralta R., Reyes-Márquez V., Franco-Sandoval L.O., Romo-Pérez A., Cruz-Navarro J.A., Mañozca-Dosman I.V., Aragón-Muriel A., Morales-Morales D. (2025). Anticancer and Antimicrobial Activity of Copper(II) Complexes with Fluorine-Functionalized Schiff Bases: A Mini-Review. Inorganics.

[43] Piccolo M., Ferraro M.G., Iazzetti F., Santamaria R., Irace C. (2024). Insight into Iron, Oxidative Stress and Ferroptosis: Therapy Targets for Approaching Anticancer Strategies. Cancers.

[44] Mariño G., Niso-Santano M., Baehrecke E.H., Kroemer G. (2014). Self-Consumption: The Interplay of Autophagy and Apoptosis. Nat. Rev. Mol. Cell Biol..

[45] Gurunathan S., Kang M.-H., Jeyaraj M., Kim J.-H. (2021). Palladium Nanoparticle-Induced Oxidative Stress, Endoplasmic Reticulum Stress, Apoptosis, and Immunomodulation Enhance the Biogenesis and Release of Exosome in Human Leukemia Monocytic Cells (THP-1). Int. J. Nanomed..

[46] Gurunathan S., Jeyaraj M., Kang M.-H., Kim J.-H. (2020). Melatonin Enhances Palladium-Nanoparticle-Induced Cytotoxicity and Apoptosis in Human Lung Epithelial Adenocarcinoma Cells A549 and H1229. Antioxidants.

[47] Liu J., Yuan C., Pu L., Wang J. (2017). Nutrient Deprivation Induces Apoptosis of Nucleus Pulposus Cells via Activation of the BNIP3/AIF Signalling Pathway. Mol. Med. Rep..

[48] Scalcon V., Bonsignore R., Aupič J., Thomas S.R., Folda A., Heidecker A.A., Pöthig A., Magistrato A., Casini A., Rigobello M.P. (2023). Exploring the Anticancer Activity of Tamoxifen-Based Metal Complexes Targeting Mitochondria. J. Med. Chem..

[49] Garrido G., Rabasa A., Sánchez B., López M.V., Blanco R., López A., Hernández D.R., Pérez R., Fernández L.E. (2011). Induction of Immunogenic Apoptosis by Blockade of Epidermal Growth Factor Receptor Activation with a Specific Antibody. J. Immunol..

[50] Kroemer G., Galluzzi L., Kepp O., Zitvogel L. (2013). Immunogenic Cell Death in Cancer Therapy. Annu. Rev. Immunol..

[51] Hoos A., Eggermont A.M.M., Janetzki S., Hodi F.S., Ibrahim R., Anderson A., Humphrey R., Blumenstein B., Old L., Wolchok J. (2010). Improved Endpoints for Cancer Immunotherapy Trials. JNCI J. Natl. Cancer Inst..

[52] Li X., Su N., Yu H., Li X., Sun S. (2024). Hainanenin-1, an Oncolytic Peptide, Triggers Immunogenic Cell Death via STING Activation in Triple-Negative Breast Cancer. Cell Commun. Signal.

[53] Wang Z., Chen Y., Fang H., Xiao K., Wu Z., Xie X., Liu J., Chen F., He Y., Wang L. (2024). Reprogramming Cellular Senescence in the Tumor Microenvironment Augments Cancer Immunotherapy through Multifunctional Nanocrystals. Sci. Adv..

[54] Xanthopoulos J.M., Romano A.E., Majumdar S.K. (2005). Response of Mouse Breast Cancer Cells to Anastrozole, Tamoxifen, and the Combination. BioMed Res. Int..

[55] Ma G., He J., Yu Y., Xu Y., Yu X., Martinez J., Lonard D.M., Xu J. (2015). Tamoxifen Inhibits ER-Negative Breast Cancer Cell Invasion and Metastasis by Accelerating Twist1 Degradation. Int. J. Biol. Sci..

