# Supplementary material for: ERα-Independent Activity of Tamoxifen-Based Transition Metal Hybrids in Triple-Negative Breast Cancer Models In Vitro and In Vivo
Source: Molecules. 2026 Apr 22;31(9):1376. doi: 10.3390/molecules31091376 (PMC13165354; doi:10.3390/molecules31091376)
Supplement: Supplementary file 1 [file molecules-31-01376-s001.zip › molecules-4221649 suppl..pdf]

Supplementary S

Supplementary S.1

Table S1. Histological assessment of the mitotic rate and necrosis percentage of tumors isolated from animals treated with L, PdL, and CuL obtained and calculated by light microscopy.

Treatment	Avg. num. of tumor cell mitoses/2mm^2	% necrosis per tumor surface
Control	39.20 ± 14.87	29.00 ± 10.24
L	36.80 ± 8.98	27.00 ± 10.37
PdL	39.20 ± 10.47	33.00 ± 7.58
CuL	32.00 ± 3.24	18.40 ± 8.76

Supplementary S.2

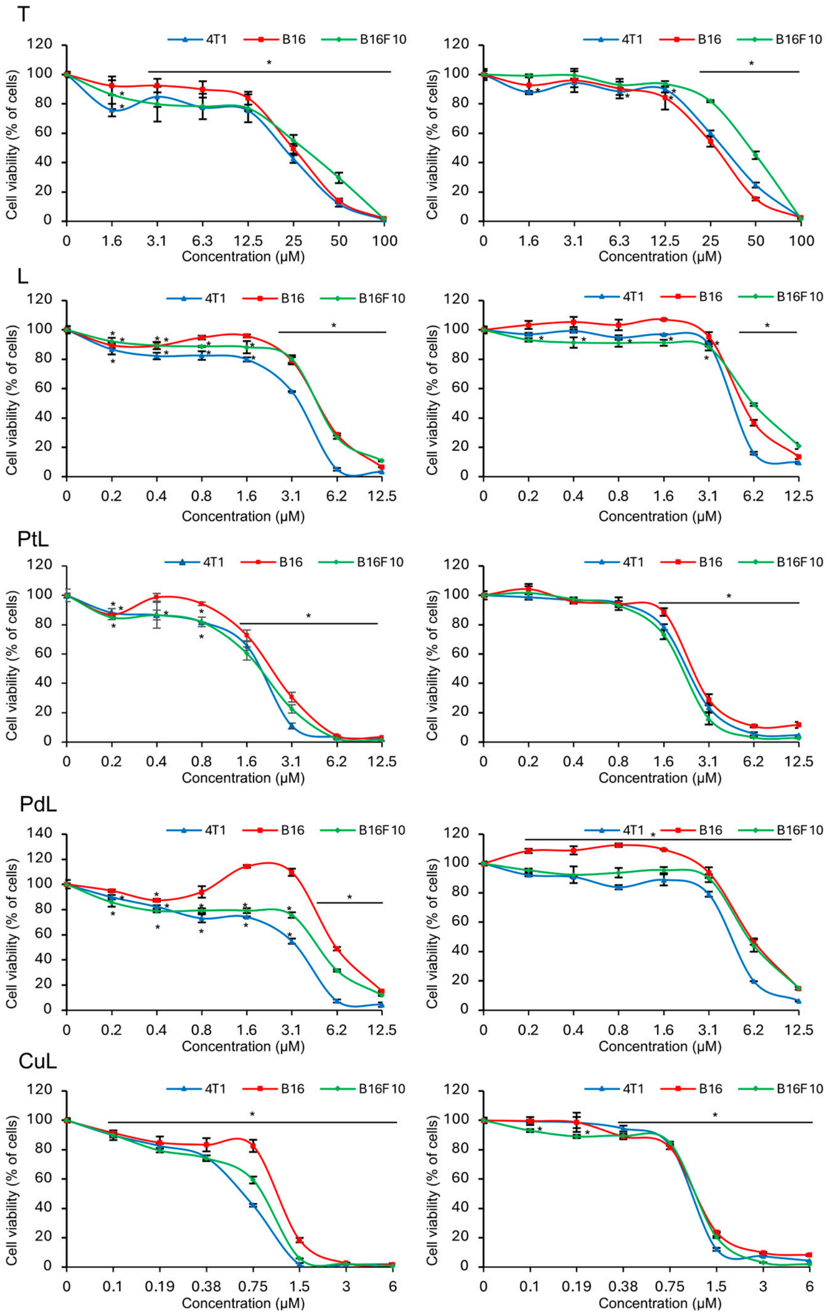

Figure S1. All experimental compounds decrease cell viability in dose-dependent manner. The viability of 4T1, B16, and B16F10 cells treated for 72 h with wide range of concentrations of experimental compounds was determined by MTT (left panel) and CV (right panel) assays. Viability was expressed as a percentage of the absorbance value of control cells that was arbitrary assigned a viability value of 100%. Data shown represent SV±SD of one representative of 3 independent experiments. **p*>0.05 compared to control

Supplementary S.3

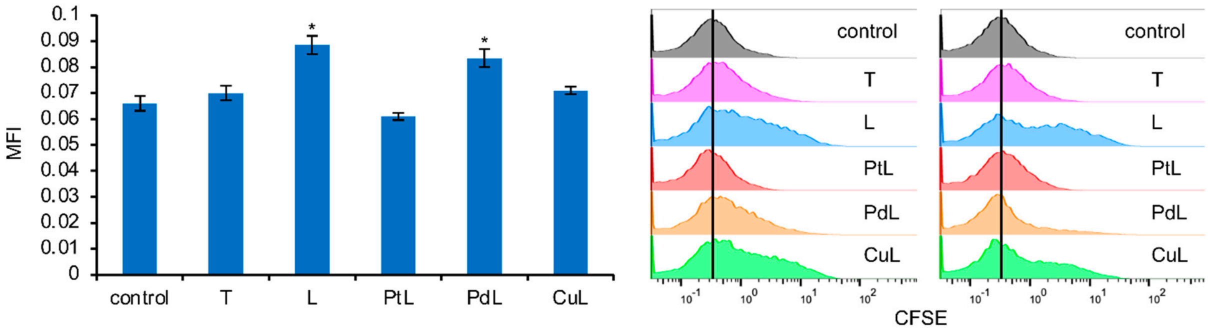

Figure S2. L and Pd-based hybrid induce moderate cell proliferation arrest. 4T1 cells were treated with IC_{50} concentrations of experimental compounds in the presence of the CFSE dye for 72 h and analyzed by flow cytometry. **p*>0.05 compared to control

Supplementary S.4

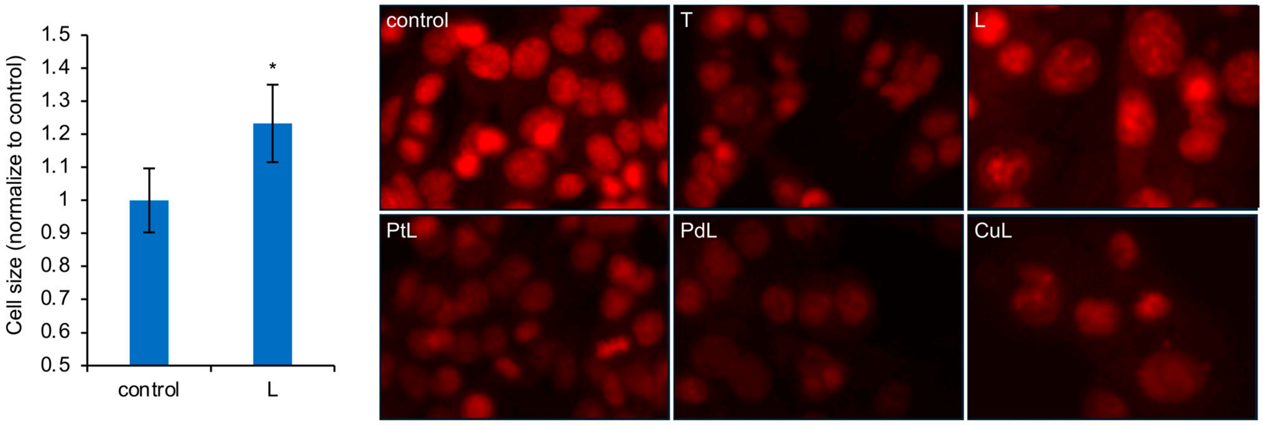

Figure S3. Ligand influences the enlargement of the cells. 4T1 were treated with IC_{50} concentrations of experimental agents for 72 h, digitally photographed by ZOE fluorescent cell imager and analyzed by photo analysis software. **p*>0.05 compared to control

Supplementary S.5

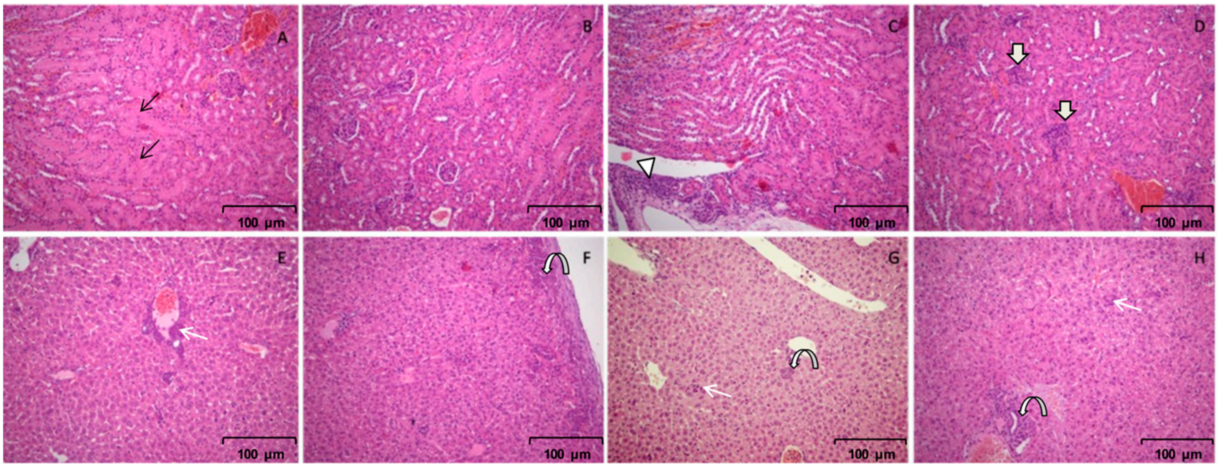

Figure S4. Histopathological resections of kidney (upper panels) and liver (lower panels) tissues isolated from animals from the control (A,E), Cu-hybrid (B,F), L (C,G), and Pd-hybrid (D,H) groups and stained with Hematoxylin and Eosin (H&E) (×200 magnification); white asterisks = areas of necrosis, black arrows = protein casts within renal tubules, white arrow = inflammatory infiltrate in the kidney, broad white arrows = periglomerular fibrosis, thin white arrows = focus of extramedullary hematopoiesis in the liver, curved arrows = focal neutrophilic infiltration
