# Supplementary figures and images for: ERα-Independent Activity of Tamoxifen-Based Transition Metal Hybrids in Triple-Negative Breast Cancer Models In Vitro and In Vivo
Source: Molecules. 2026 Apr 22;31(9):1376. doi: 10.3390/molecules31091376 (PMC13165354; doi:10.3390/molecules31091376)

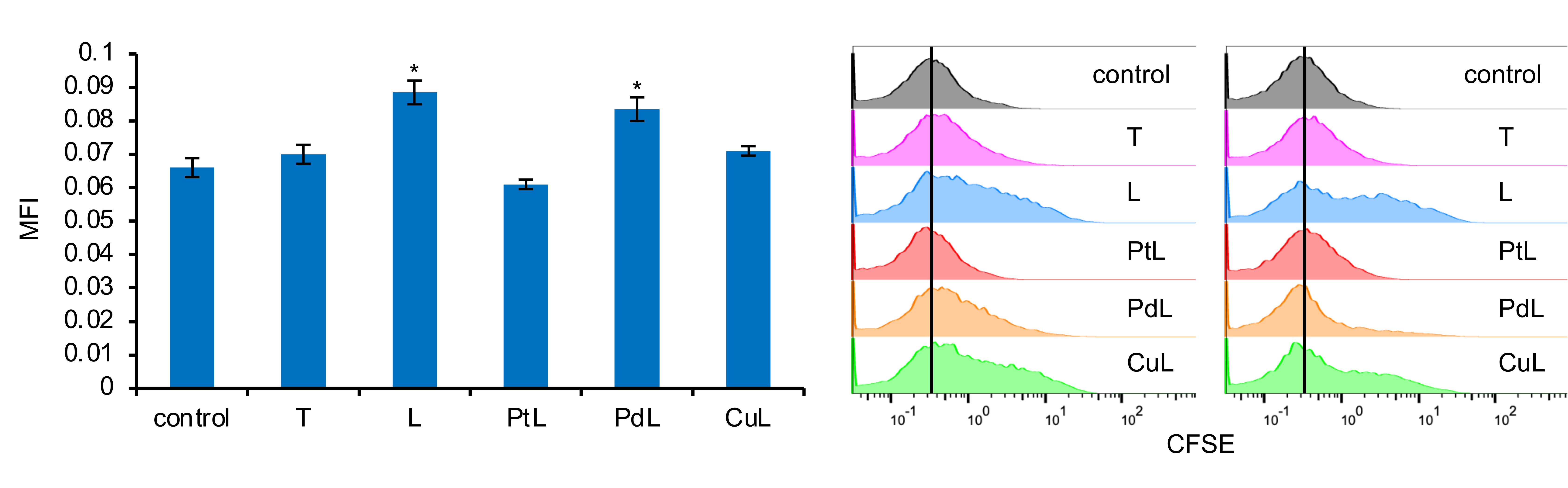

Supplement: Supplementary file 1 [file molecules-31-01376-s001.zip › Figure S2.tiff]

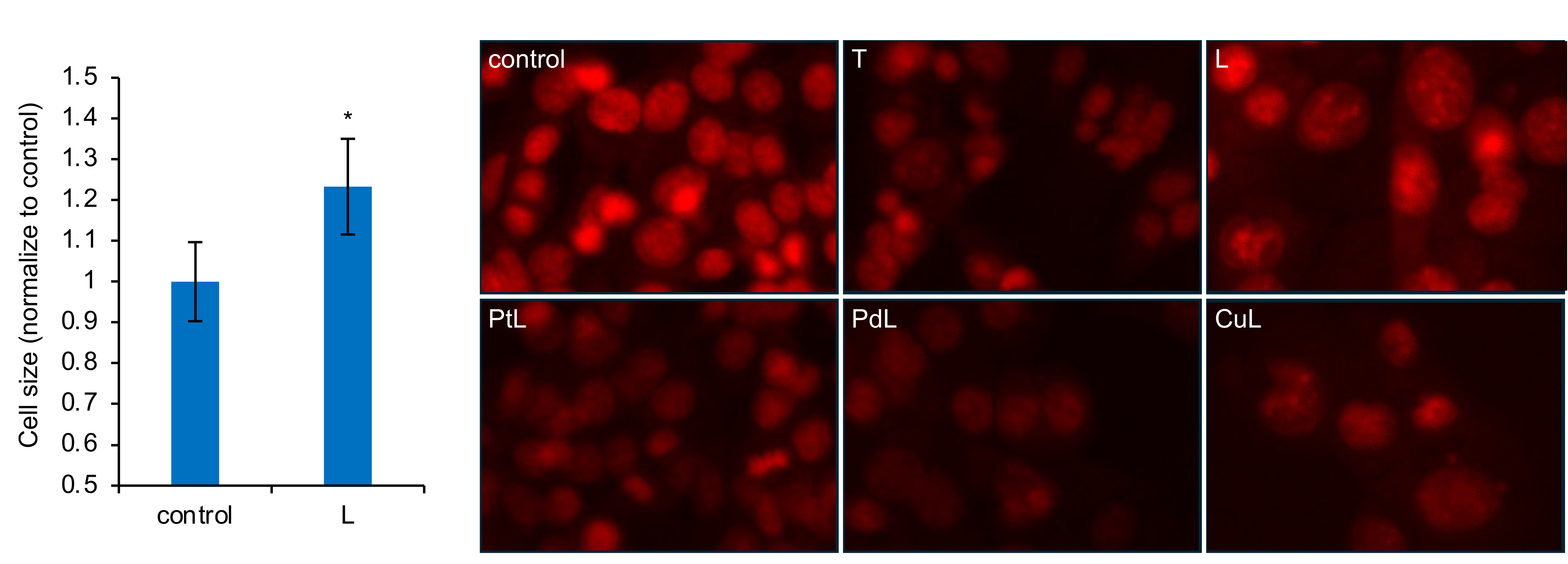

Supplement: Supplementary file 1 [file molecules-31-01376-s001.zip › Figure S3.tiff]

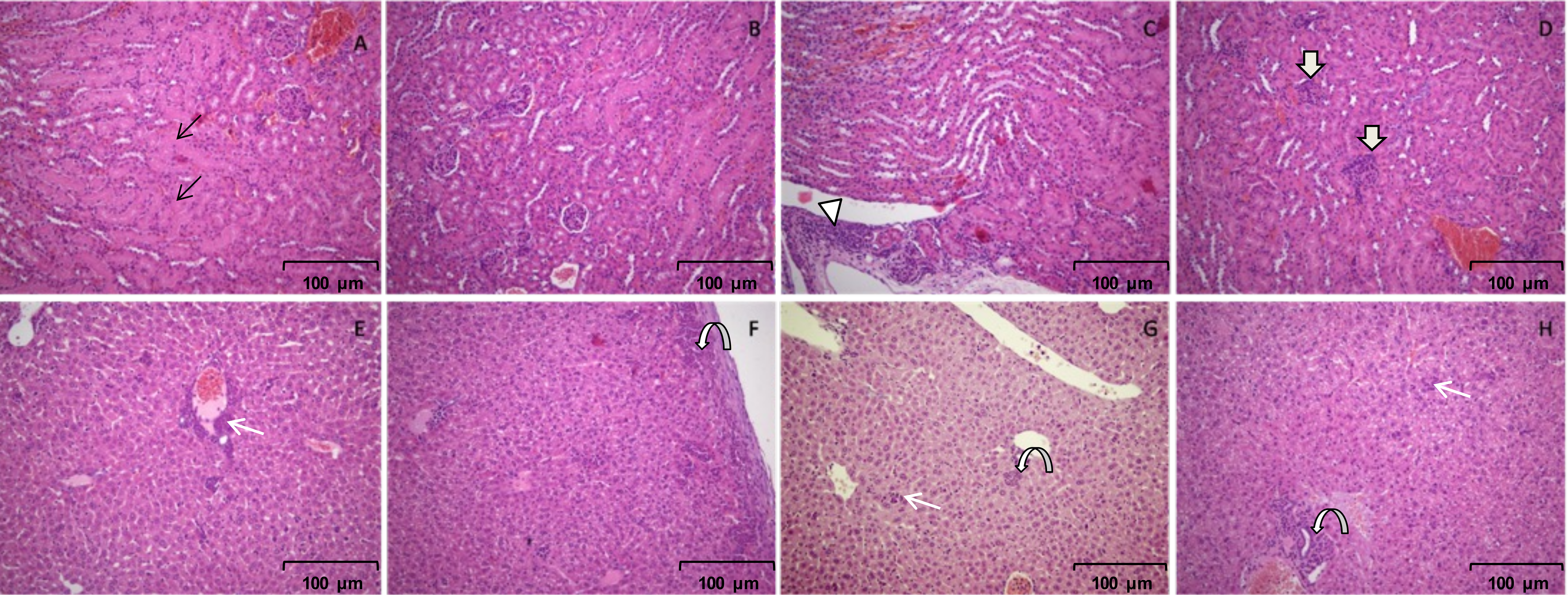

Supplement: Supplementary file 1 [file molecules-31-01376-s001.zip › Figure S4.tiff]
